# Long-term functional outcome after laryngeal cancer treatment

**DOI:** 10.1186/s13014-019-1299-8

**Published:** 2019-06-11

**Authors:** Lukas Anschuetz, Mohamed Shelan, Marco Dematté, Adrian D. Schubert, Roland Giger, Olgun Elicin

**Affiliations:** 1 Department of Otorhinolaryngology, Head & Neck Surgery, Inselspital, Bern University Hospital, University of Bern, 3010 Bern, Switzerland; 2 Department of Radiation Oncology, Inselspital, Bern University Hospital, University of Bern, 3010 Bern, Switzerland; 3 Department of Otorhinolaryngology, Head & Neck Surgery, Head and Neck and Sensory Organs Department, Sant’Orsola-Malpighi Hospital, University of Bologna, 40138 Bologna, Italy

**Keywords:** Laryngeal cancer, Tracheostomy, Feeding tube, Outcome, Functional outcome, Recurrence, Quality of life

## Abstract

**Background:**

The functional outcome after the treatment of laryngeal cancer is tightly related to the quality of life of affected patients. The aim of this study is to describe the long-term morbidity and functional outcomes associated with the different treatment modalities for laryngeal cancer.

**Methods:**

Retrospective chart review of 477 patients undergoing curatively intended treatment for laryngeal cancer at our tertiary referral center from 2001 to 2014: Details on patient and disease characteristics, diagnostics and treatment related functional outcomes were analyzed.

**Results:**

With a median follow-up of 51 months, the crude rate of functional larynx preservation was 74.6%. Radiotherapy +/− chemotherapy was the dominant treatment modality (*n* = 359–75.3%), whereas 24.7% (*n* = 118) underwent primary surgery, with 58.5% (69) receiving adjuvant treatment. The 5-year laryngectomy-free survival was 57% (95% CI, 48–66%) after surgery vs. 69% (95% CI, 64–75%) after chemoradiotherapy (*p* < 0.01). In stage III-IVB, these rates were 26% (95% CI, 16–39%) vs. 47% (95% CI, 36–59%), respectively (*p* < 0.01). Aspiration occurred in 7%, tracheostomy was necessary in 19.8% and feeding tube placement in 25.4%. Feeding tube and tracheostomy necessity was higher in the initially surgically treated group. Primary surgery (HR: 1.67, 95% CI: 1.19–2.32; *p* < 0.01), stage III-IVB (HR: 4.07, 95% CI: 2.97–5.60; p < 0.01) and tumor recurrence (HR: 3.83, 95% CI: 2.79–5.28; *p* < 0.01) remained as adverse factors for laryngectomy-free survival.

**Conclusions:**

Preserving the laryngeal function after cancer treatment is challenging. Advanced tumor stages, primary surgery and recurrence are related to a poor functional outcome. Therefore, the criteria for initial decision-making needs to be further refined.

**Electronic supplementary material:**

The online version of this article (10.1186/s13014-019-1299-8) contains supplementary material, which is available to authorized users.

## Background

Squamous cell carcinoma (SCC) of the larynx is a challenging entity with different treatment strategies and related functional outcomes. The assessment of therapy outcome is hindered by the considerable heterogeneity of initial disease presentation, as well as possible debilitating functional impairments related to the tumor and/or its treatment [1]. Laryngeal functions such as breathing, phonation and swallowing are crucial to the patients’ quality of life and therefore require special consideration.

Radiotherapy +/− concomitant chemotherapy ((C)RT) with organ-preservation protocols have been adopted during the last three decades to keep the structural and functional integrity of the larynx intact [2, 3]. Nevertheless, the damage to healthy tissues through (C)RT is considerable. Abundant therapy-related toxicity poses substantial problems [4]. Long-term sequelae of radiation-induced toxicity include tissue fibrosis, which may lead to a hypo- or immobility of the laryngeal subsites like vocal folds, cricoarytenoid joints and aryepiglottic folds. Additional chronic mucosal swelling, damage to constrictor muscles and potentially occurring chondroradionecrosis may lead to further impaired laryngo-pharyngeal function (e.g. airway obstruction, hoarseness, stenosis and aspiration). These may lead to an impaired quality of life, especially in loco-regionally advanced tumor stages [5]. A recent analysis identified a high number of feeding tube (FT) and tracheostomy dependency in head and neck cancer patients treated by (C)RT [6]. Therefore, the important question regarding the outcome after curatively intended treatment of laryngeal cancer concerns not only the preservation of the larynx, but also the preservation of its function.

The aim of this study is to report 1) the long-term sequelae and functional outcomes at last follow-up; 2) laryngectomy-free survival; and to 3) identify the factors associated with poor functional outcome for early- and advanced-stage laryngeal cancer at our tertiary reference center.

## Methods

The paper and electronic records of all patients presented with laryngeal SCC at our tertiary referral center’s head and neck tumor board between 2001 and 2014 were reviewed (*n* = 594). A total of 117 patients were excluded due to the following reasons: non-curatively intended treatment (*n* = 52), presence of any other synchronous or previous malignant tumor (even if successfully treated at the time of laryngeal SCC diagnosis, *n* = 55), or incomplete documentation (*n* = 10). Finally, 477 patients with accomplished curatively intended treatment were included in the present study.

Patient and disease characteristics were assessed and staged with the Union for International Cancer Control (UICC) 7th edition. The treatment associated immediate, early and late complications, recurrence patterns, corresponding treatments after recurrence and the functional long-term outcome at the last follow-up were assessed. “Functional larynx” was defined as an intact larynx enabling serviceable voice without aspiration, tracheostomy and/or FT. The dates of death, tumor recurrence and laryngectomy were available throughout the whole cohort. However, it was not possible to fully acquire the exact time points and durations of all complications: installation and removal of FTs, opening and closure of tracheostomies, beginning and end of aspiration etc. Therefore, these were classified as transient and permanent (i.e. until death or last follow-up) in the database. Events for laryngectomy-free survival (LFS) were defined as laryngectomy or death, regardless of the reason (functional, recurrence or upfront within the frame of primary surgical treatment). The time-to-event outcomes were calculated based on the date of diagnosis, and evaluated by Kaplan-Meier curves and log-rank test. Chi-square test was used to compare categorical variables. In order to isolate the adverse factors influencing the functional outcome parameters, multivariate Cox proportional hazard and nominal logistic models with variables yielding *p* values < 0.1 via univariate analyses were built, and backwards elimination was performed. Statistical analysis was done with JMP software (version 13.0 SAS Institute, Cary, NC, USA). Statistical significance was set to a two-tailed alpha of < 0.05. The actuarial rates and risk estimations are provided with 95% confidence intervals (CI).

## Results

A total of 477 patients were eligible for the present study after curatively intended treatment for laryngeal cancer in our institution. The median follow-up of surviving patients was 51 months. The patients’ demographic and disease characteristics are reported in Table 1.

**Table 1:** Patient and disease characteristics

Parameter	n (%)
Total	477 (100%)
Male sex	428 (89.7%)
Median Age (range)	64 (36–92)
Tumor subsite	
Supraglottic	124 (26%)
Glottic	281 (58.9%)
Subglottic	13 (2.7%)
Transglottic	59 (12.4%)
cT stage	
1a	185 (38.8%)
1b	47 (9.9%)
2	118 (24.7%)
3	79 (16.6%)
4a	47 (9.9%)
4b	1 (0.2%)
cN stage	
0	388 (81.3%)
1	24 (5%)
2a	4 (0.8%)
2b	31 (6.5%)
2c	24 (5%)
3	6 (1.3%)
Clinical UICC stage	
I	226 (47.4%)
II	97 (20.3%)
III	65 (13.6%)
IVA	82 (17.2%)
IVB	7 (1.5%)

*UICC* Union for International Cancer Control (7th edition)

The predominant primary treatment modality was (C)RT (75.3% - *n* = 359/477), whereas 24.7% (*n* = 118/477) underwent primary surgery. The features and distribution of treatment modalities are provided in Table 2. The 5-year overall survival was 79%.

**Table 2:** Features and distribution of treatment modalities

Treatment Parameters	Primary Treatment Modality	
	Surgery	(Chemo)Radiotherapy
	*n* = 118 (24.7%)	*n* = 359 (75.3%)
Transoral laser microsurgery	62 (52.5%)	
Partial open laryngectomy	17 (14.4%)	
Total laryngectomy	39 (33.1%)	
Neck Dissection	62 (52.5%)	25 (7%)^a^
Adjuvant treatment	69 (58.5%)	
Radiotherapy technique		
conventional 2-dimensional	8 (11.6%)	9 (2.5%)
conventional 3-dimensional	47 (68.1%)	230 (64.1%)
static field intensity modulated	10 (14.5%)	48 (13.4%)
volumetric modulated arc	4 (5.8%)	72 (20.1%)
Median total dose in Gy (range)	66 (60–72)	70 (64–76)
Median duration of radiotherapy in days (range)	46 (36–59)	49 (39–71)
Induction chemotherapy	2 (1.7%)	9 (2.5%)
Concomitant systemic therapy	19 (27.5%)	92 (25.6%)
Cisplatin 3w	15 (78.9%)	61 (66.3%)
Carboplatin + 5-fluorouracil 3w	2 (10.5%)	7 (7.6%)
Cetuximab weekly	2 (10.5%)	24 (26.1%)

^a^ up-front neck dissection followed by primary chemoradiotherapy, *3w* Three-weekly regimen

### Risk of total laryngectomy

**Fig. 1:**
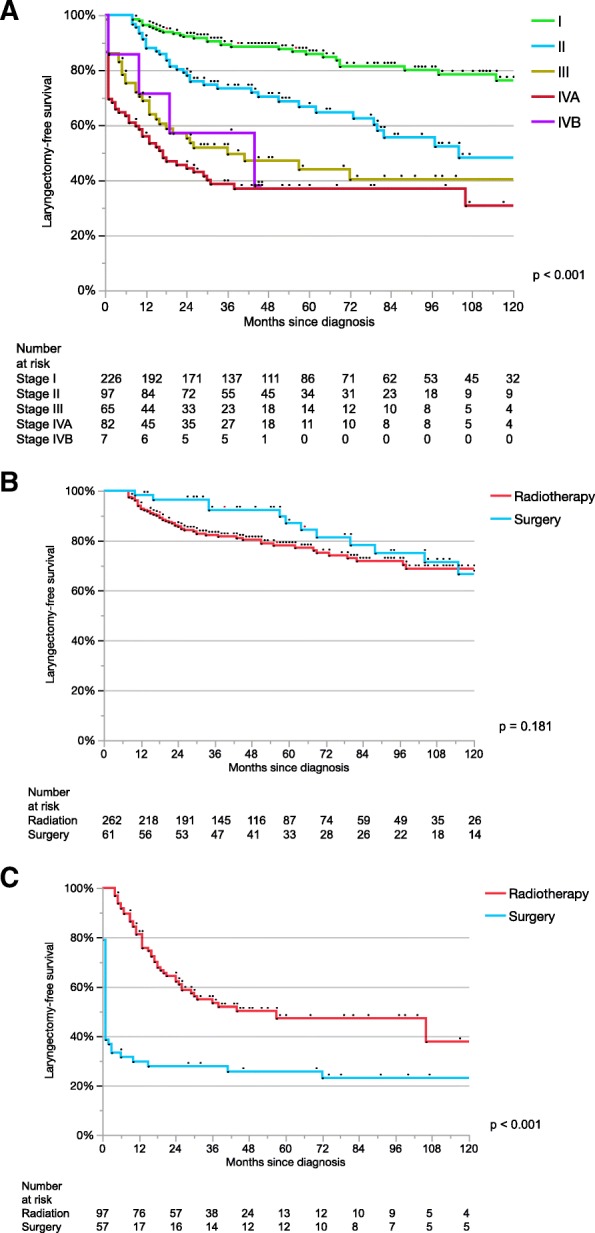
Laryngectomy-free survival by stage and primary treatment modality. Laryngectomy-free survival based on clinical stage (**a**); primary treatment modality in early stage (**b**); primary treatment modality in locally-advanced stage

In total, 100 patients (21%) underwent TL. The 5-year LFS was 57% (95% CI, 48–66%) vs. 69% (95% CI, 64–75%) with primary surgery vs. primary (C)RT, respectively. Figure 1a depicts the LFS distributed for UICC stage. When the stages were grouped as early (I-II) and locally-advanced (III-IVB), both primary treatment modalities offered similar LFS rates in early stages (Fig. 1b), whereas non-surgical treatment offered a statistically significant higher chance to preserve the larynx in locally-advanced stages (47% (95% CI, 36–59%) vs. 26% (95% CI, 16–39%), *p* < 0.01) (Fig. 1c). However, this difference was rather caused by the number of patients treated with upfront TL in the surgical group, and not due to salvage or functional TL after (C)RT (2 patients). In the functional TL group, the first patient had a cT4a cN1 disease, initially treated with induction chemotherapy followed by concomitant (C)RT. She suffered from life-threatening laryngeal stenosis and aspiration caused by chondroradionecrosis. The second patient was diagnosed with a cT1a cN0 glottic laryngeal cancer treated with transoral laser surgery. After 6 years, he underwent salvage (C)RT due to recurrence, which subsequently caused a chondroradionecrosis leading to laryngeal stenosis requiring a functional TL.

### Risk of tracheostomy

Having tracheostomy at any time point was observed in 53.4% (*n* = 63/118) and 29.5% (106/359) patients who were primarily treated with surgery (including TL) and (C)RT, respectively (*p* < 0.01). Similarly, the rate of tracheostomy at the last follow-up was higher in patients who underwent primary surgical treatment compared to patients who were initially treated with (C)RT (38.9% (*n* = 46/118) vs. 20.9% (*n* = 75/359), p < 0.01). These differences were more prominent in the locally-advanced tumors: tracheostomy at any time point (87.7% (*n* = 50/57) vs. 51.6% (n = 50/97), *p* < 0.01), tracheostomy at the last follow-up (70.2% (*n* = 40/57) vs. 33% (*n* = 32/97), p < 0.01). However, when excluding patients who initially underwent TL, we observed a similar rate for tracheostomy at any time point in surgically treated patients of 29.5% (*n* = 106/359) and of 30.4% (*n* = 24/79) in (C)RT patients (*p* = 0.881), but a lower rate of tracheostomy at the last follow-up was observed in surgically treated patients (8.9% - *n* = 7/79) than (20.9% - *n* = 75/359) in (C)RT patients (*p* < 0.01).

### Risk of feeding tube

Having a FT at any time point was 51.7% (*n* = 61/118) and 30.4% (*n* = 109/359) in primary surgery and (C)RT groups, respectively (p < 0.01). These ratios were distributed as 18% (*n* = 58/323) and 72.7% (*n* = 112/154) in stages I-II and III-IVB, respectively (p < 0.01). However, the rate of being dependent on a permanent FT was relatively low: 3.4% (*n* = 4/118) after primary surgery, 5% (*n* = 18/359) after primary (C)RT, *p* = 0.45. Again, after exclusion of patients treated initially with TL, the rate of a FT at any time point was 29.1% (*n* = 23/79) in the surgical and 30.4% (n = 109/250) in the (C)RT group (*p* = 0.826), and the rate of being dependent on a FT at the last follow-up was 1.3% (n = 1/79) after initial surgery and 5% (n = 18/359) after initial (C)RT (*p* = 0.092).

### Early and late complications

Two patients died within 30 days of treatment (0.4%). The following additional rates of complications were observed: pharyngeal stenosis (4.6% - *n* = 22/477), laryngeal stenosis (24.7% - *n* = 108/438), chondroradionecrosis (2.5% - *n* = 11/438) and soft tissue necrosis (4.4% - *n* = 21/477). The rates and corresponding time intervals of aspiration pneumonias, tracheostomies and FTs are shown in Table 3.

**Table 3:** Time intervals (time of occurrence after end of treatment) of aspiration pneumonias, tracheostomies and feeding tubes

Time Interval^a^	Aspiration^b^ (permanent)	Tracheostomy^b^ (permanent)	Feeding Tube (permanent)
Any	7% (1.8%)	19.8% (3.5%)	25.4% (3.5%)
Peri-treatment	3.4% (0.7%)	10% (1.4%)	20.5% (2.5%)
3 to 24 months	2.7% (0.2%)	7.3% (1.6%)	3.6% (0.8%)
After 2 years	0.9% (0.9%)	2.5% (0.5%)	1.3% (0.2%)

^a^ Events after tumor recurrence (73) are censored

b Patients who initially underwent total laryngectomy (*n* = 39) are excluded

A functional larynx at last follow-up was obtained in 74.6% (*n* = 356/477) in the whole cohort, whereas in 84.8% (*n* = 274/323) and 53.2% (*n* = 82/154) in early and locally-advanced stages, respectively (*p* < 0.01). These rates were 79.1% (*n* = 284/359) and 61% (*n* = 72/118) with primary (C)RT and surgery, respectively (p < 0.01).

### Outcome after salvage treatment

We observed a tumor recurrence in 28.7% (*n* = 137/477) of patients. Of those, 99 cases underwent a salvage treatment with curative intent. In this context, 55 TL and 23 tracheostomies were performed. One patient died within 30 days after salvage treatment. The following complications developed after salvage treatment: permanent FT requirement (35% - *n* = 35/99), soft tissue necrosis (19% - *n* = 19/99), aspiration pneumonia (18% - *n* = 18/99), pharyngeal stenosis (15% - *n* = 15/99), laryngeal stenosis (8% - *n* = 8/99) and chondroradionecrosis (2% - *n* = 2/99).

### Multivariate analysis

Locally-advanced tumor stage, primary surgery, and tumor recurrence remained as adverse factors for permanent tracheostomy (Additional file 1: Table S1) and LFS (Table 4). Tumor subsite as a possibly influencing variable was not included in the multivariate models, because the origin of the tumor could not be determined and was categorized as transglottic in 59 cases (Table 1). Tumor stage remained as adverse factor for permanent FT dependency (Additional file 1: Table S1).

**Table 4:** Multivariate model for laryngectomy-free survival

Variable	HR (95% CI)	*p* value
Age > 64 vs. ≤64	1.35 (0.99–1.85)	0.060
Female vs. male	0.85 (0.47–1.41)	0.540
Stage III-IV vs. I-II	4.29 (3.11–5.93)	< 0.001*
Primary surgery vs. (C)RT	1.84 (1.30–2.57)	< 0.001*
Tumor recurrence vs. not	3.83 (2.79–5.28)	< 0.001*

*CI* Confidence interval, *(C)RT* (Chemo)radiation, *HR* Hazard ratio

* Remained *p* < 0.05 after backwards elimination

Advanced tumor stage (cT), primary surgery and tumor recurrence were identified as adverse factors regarding laryngeal function (as defined above) (summarized in Table 5).

**Table 5:** Multivariate model for non-functional larynx at the time of last follow-up

Variable	OR (95% CI)	*p* value
Age > 64 vs. ≤64	0.90 (0.54–1.50)	0.688
Female vs. male	0.83 (0.35–1.98)	0.669
cT 3–4 vs. 1–2	10.19 (5.54–18.73)	< 0.001*
cN 2–3 vs. 0–1	0.54 (0.27–1.10)	0.092
Primary surgery vs. (C)RT	2.45 (1.42–4.22)	0.001*
Tumor recurrence vs. not	11.16 (6.36–19.58)	< 0.001*

*CI* Confidence interval, *(C)RT* (Chemo)radiation, *OR* Odds ratio

* Remained *p* < 0.05 after backwards elimination

## Discussion

In this study, we report long-term functional outcome after curatively intended laryngeal SCC treatment of all tumor stages. The 5-year LFS was 57% (95% CI, 48–66%) vs. 69% (95% CI, 64–75%) with primary surgery vs. primary (C)RT, respectively (*p* < 0.01). A functional larynx was observed in 74.6% of the patients at the time of last follow-up. Of those, 1.4% remained dependent on FT. Overall, aspiration was observed in 7% (1.8% permanent), tracheostomy in 19.8% (3.5% permanent) and FT dependency in 25.4% (3.5% permanent) of the whole cohort within all stages. The multivariate model identified primary TL, advanced stages and recurrence as adverse effects on LFS.

Laryngeal functions such as the protection of the airway during breathing and swallowing are crucial for the patients’ quality of life and are threatened by laryngeal cancer and the performed treatments. Total laryngectomy is separating the airway from the pharynx and esophagus, and therefore no natural function can be preserved. In contrast, organ-preservation strategies aim to preserve the natural anatomy and the function of the larynx, whilst offering similar oncological results. As reported in the Veterans Affairs trial, which was conducted with a prospective assessment of swallowing and voice, a significant difference regarding speech intelligibility between the TL and the (C)RT group after 2 years of follow-up was observed, favoring organ-preservation concerning better communication. However, no difference regarding swallowing was reported between the two treatment modalities. Surprisingly, no patients were reported using a percutaneous FT [7, 8]. Our cohort has a high prevalence of tracheostomy in patients undergoing primary surgical therapy (53.4%). This is mainly related to cases of primary TL and prophylactic tracheostomies in cases of partial laryngectomies. Of these patients, 39% ended up with a permanent tracheostomy. In the (C)RT group, tracheostomy was observed in 29%, of which 21% remained permanent (including salvage TL). Tracheostomy and/or TL was associated to locally-advanced tumors or salvage treatment for recurrence. We did not observe other statistically significant correlations, probably because we report all tumor stages and therefore studied a heterogeneous cohort regarding tumor extension.

Actually, most of the literature dealing with tracheostomies in the context of laryngeal cancer include also hypopharyngeal cancers. However, we think that these tumors should be assessed separately. According to the literature reporting only results of laryngeal SCC, the necessity of a tracheostomy varies between 8.2 and 25% [9–11]. Factors related to permanent tracheostomies are pre-treatment tracheostomy, extension of disease (poor laryngeal function prior to treatment), post-irradiated planned or salvage neck dissection and primary RT in the context of organ-preservation protocols [12–15]. Moreover, it has been discussed in the literature, whether the presence of long-term tracheostomy influences the overall survival, but apparently the 5-year survival is not influenced [13].

Low aspiration rates of 7% (1.8% permanent) in the long-term follow-up were assessed in the present study. Comparing to other studies reporting higher aspiration rates, we have to consider that they also investigated asymptomatic patients [16–19]. In contrast, our data may underestimate the prevalence of subclinical swallowing disorders or aspiration since instrumental swallowing studies were only performed when patients complained of dysphagia.

In this cohort, a high prevalence of initial FT placement (51.7 and 30.4% in primary surgery and (C)RT group, respectively) was observed. We explain this high incidence of enteral feeding at any time point in surgically treated patients by the short-term enteral nutrition usually required during wound healing in the immediate post-operative course. Similarly, the high prevalence of FT in the primary (C)RT is related to our former policy to regularly offer prophylactic percutaneous FT placement in loco-regionally advanced tumor stages. In the long-term outcome, we observed generally a good swallowing function with a low rate of permanent FT dependency (3.4% after primary surgery, 5% after primary (C)RT). Therefore, the prophylactic percutaneous FT placement in (C)RT patients appears to be obsolete. Acute problems with oral feeding related to (C)RT acute toxicity could be resolved by the placement of a nasogastric FT. Regarding Lefebvre et al. (2009), a FT has been placed in 43 patients and was left in place for more than 3 months in 40 patients in the sequential arm (18%) of the EORTC 24954 trial. Similarly, a FT was placed in 39 patients and left in place for more than 3 months in 35 patients in the alternating arm (15%) [9]. Wopken et al. (2018) reported in a systematic review of the literature a high variability of FT dependency over time. Essentially, after 2 years, the rate varied from 3.7 to 10% [20]. Regarding partial laryngectomies, a recent systematic review of the literature regarding dysphagia following supracricoid laryngectomy, reported a complete FT dependency in 0.7% [21]. Weinstein et al. (2007) observed in a cohort of primary and salvage supracricoid laryngectomies a good swallowing function with only 6% of the patients requiring a FT [22].

The latest American Society of Clinical Oncology consensus guidelines recommend for T1 and T2 laryngeal carcinomas either transoral resection or RT alone, whereas for “early” T3 and T4, either organ-preservation surgery or RT or (C)RT [23]. However, for extensive T3, large T4a destructing the cartilages of larynx or poor pretreatment laryngeal function, TL is advised [23]. Our results showing a statistically significant adverse functional outcome after the treatment of advanced tumors support these recommendations. The primary surgical treatment with consecutive separation of the airway from the upper digestive tract may resolve many issues related to a poorly functioning larynx, especially aspiration and swallowing deficits. On the other side, the functional outcome regarding upfront TL is significantly inferior according to our results. Therefore, indications for an initial surgical approach have to be carefully evaluated. However, we have to bear in mind that salvage surgery after (C)RT failure is prone to frequent local and systemic complications of up to 63% [24–27].

The main limitation of this study is obviously its retrospective design. Therefore, we were not able to quantitatively report graded long-term toxicity data. Moreover, the assessment of speech, social and emotional outcomes was not possible from the documentations. Another shortcoming was the lack of exact end dates of all complications. Finally yet importantly, our cohort is subject to selection bias due to its retrospective nature. First of all, there is a clear imbalance between the numbers of patients treated with primary (C)RT and surgery. A concerning rate of the surgically treated patients with early stage disease underwent adjuvant RT instead of re-resection. This may be explained by our previous institutional policy apparently favoring RT over surgery in early stage larynx cancer, not only as the first treatment option but also in case of close and positive resection margins, which underwent a major change few years ago.

An interesting aspect not often taken into consideration in the scientific debate on optimal treatment options is the patient’s will. It is the duty of the treating multidisciplinary team to evaluate and offer the best treatment option(s) to the patients. Functional results and their impact on the patient’s quality of life play an important role in counseling the patient. This aspect is a strength of the present study, reporting the long-term “real-life” situation in a tertiary referral center. It is our philosophy to offer the patient different treatment options, including our opinion about the best treatment. Finally, the patient should be able to make the informed decision and have the possibility to trade off chance of survival against treatment related morbidity and adverse effects. There are not many studies [28, 29] describing and analyzing the patient’s will in relation to laryngeal cancer treatment. Hence, this is an interesting subject, since laryngeal cancer may be treated in different ways. The most recent study by Laccourreye et al. (2014) suggest that: (i) laryngeal preservation is not a primary objective shared by all subjects; (ii) the percentage chance of cure that subjects would be willing to trade off to avoid total laryngectomy is extremely variable and (iii) the information on the major functional risks inherent to laryngeal preservation protocols has a major impact on the subject’s treatment decision [30]. Therefore, we have to encourage the scientific community to continuously monitor and compare functional treatment outcomes. It is the duty of the multidisciplinary team in a comprehensive head and neck cancer center not only to offer best therapy options, but also to adequately counsel patients on different treatment strategies and related sequelae.

## Conclusions

In this study with a fair median follow-up time, a full conservation of laryngeal function at the last follow-up in 74.6% of patients undergoing curatively intended treatment for laryngeal SCC of all stages is reported. Advanced tumor stages, primary surgery and recurrent disease are related to a poor functional outcome. Preserving the laryngeal function in laryngeal cancer treatment is challenging. The criteria for initial decision-making needs to be refined.

## Additional file

Additional file 1: **Table S1.** Multivariate model for permanent tracheostomy and feeding tube dependency. (DOCX 13 kb)

## Data Availability

Anonymized version of the data is available upon reasonable request.

## Abbreviations

(C)RT: Radiotherapy +/− concomitant chemotherapy
CI: Confidence interval
FT: Feeding tube
HR: Hazard ratio
LFS: Laryngectomy-free survival
OR: Odds ratio
SCC: Squamous cell carcinoma
TL: Total laryngectomy
UICC: Union for International Cancer Control
