# Supplementary material for: Long-term functional outcome after laryngeal cancer treatment
Source: Radiat Oncol. 2019 Jun 11;14:101. doi: 10.1186/s13014-019-1299-8 (PMC6558792; doi:10.1186/s13014-019-1299-8)
Supplement: Supplementary file 1 — Table S1. Multivariate model for permanent tracheostomy and feeding tube dependency. (DOCX 13 kb) [file 13014_2019_1299_MOESM1_ESM.docx]

Additional file 1: **Table S1** Multivariate model for permanent tracheostomy and feeding tube dependency

| **Variable** | **OR (95% CI) for permanent tracheostomy** | **p value** | **OR (95% CI) for permanent feeding tube** | **p value** |
| --- | --- | --- | --- | --- |
| Age >64 vs. ≤64 | 1.09 (0.66-1.78) | 0.741 | 1.56 (0.64-3.77) | 0.327 |
| Female vs. male | 0.82 (0.35-1.92) | 0.642 | 0.97 (0.21-4.41) | 0.965 |
| Stage III-IV vs. I-II | 5.74 (3.41-9.64) | <0.001* | 3.44 (1.41-8.43) | 0.007* |
| Primary surgery vs. (C)RT | 2.46 (1.45-4.17) | <0.001* | 1.83 (0.59-5.70) | 0.295 |
| Tumor recurrence vs. not | 9.42 (5.54-16.01) | <0.001* | 1.93 (0.79-4.70) | 0.150 |

CI: confidence interval; (C)RT: (chemo)radiation; OR: odds ratio

* Remained p<0.05 after backwards elimination
